# Functional insights into the interplay between DNA interaction and metal coordination in ferric uptake regulators

**DOI:** 10.1038/s41598-018-25157-6

**Published:** 2018-05-08

**Authors:** Sabina Sarvan, François Charih, Momen Askoura, James Butcher, Joseph S. Brunzelle, Alain Stintzi, Jean-François Couture

**Affiliations:** 1 Ottawa Institute of Systems Biology, Department of Biochemistry, Microbiology and Immunology, University of Ottawa, 451 Smyth Road, Ottawa, Ontario K1H 8M5 Canada; 2 Department of Microbiology and Immunology, Faculty of Pharmacy, Zagazig University, Zagazig, Egypt; 3 Feinberg School of Medicine, Department of Molecular Pharmacology and Biological Chemistry, Northwestern University, Chicago, Illinois 60611 USA

## Abstract

Ferric uptake regulators (Fur) are a family of transcription factors coupling gene regulatory events to metal concentration. Recent evidence has expanded the mechanistic repertoires employed by Fur to activate or repress gene expression in the presence or absence of regulatory metals. However, the mechanistic basis underlying this extended repertoire has remained largely unexplored. In this study, we used an extensive set of mutations to demonstrate that *Campylobacter jejuni* Fur (CjFur) employs the same surface to positively and negatively control gene expression regardless of the presence or absence of metals. Moreover, the crystal structure determination of a CjFur devoid of any regulatory metals shows that subtle reorientation of the transcription factor DNA binding domain negatively impacts DNA binding, gene expression and gut colonization in chickens. Overall, these results highlight the versatility of the CjFur DNA binding domain in mediating all gene regulatory events controlled by the metalloregulator and that the full metalation of CjFur is critical to the *Campylobacter jejuni* life cycle *in vivo*.

## Introduction

Transition metals are crucial components of several metabolic pathways and are critical for DNA, RNA and protein synthesis^1,2^. However, when found in excess, these metal ions are toxic. To prevent toxicity and control their intracellular availability, metal ion concentrations are tightly controlled^3,4^. In bacteria, a large group of metal-sensing transcription factors regulates the intracellular homeostasis of metal cofactors. These metalloregulators are divided into seven different families that are important for the detection of the six biologically essential transition metals, Mn^II^, Fe^II^, Co^II^, Ni^II^, Cu^I^ and Zn^II^, as well as other transition metals such as Ag, Au, Cd^II^ and Hg^II ^^3^. One family, the ferric uptake regulator (Fur) family of metalloregulators, is involved in the regulation of iron, manganese, zinc and nickel homeostasis through the activity of Fur, Mur (manganese uptake regulator), Zur (zinc uptake regulator) and Nur (nickel uptake regulator) proteins, respectively. In addition to these members, the Fur family of metalloregulators also includes the peroxide stress regulator (PerR) and the heme-dependent iron responsive regulator (Irr)^4–11^. Depending on the availability of metal cofactors, the Fur superfamily regulates the expression of proteins involved in metal acquisition, storage and consumption in order to maintain metal homeostasis and ensure that the concentration of free metals do not reach toxic levels^2^. In addition, the Fur family of transcription factors regulates the expression of mRNAs encoding for proteins implicated in energy metabolism, acid and oxidative stress defense, the tricarboxylic acid cycle, protein glycosylation and flagella biogenesis^12,13^. Moreover, members of the Fur superfamily play a role in the expression of virulence factors in most pathogens^4^ and are therefore important factors contributing to bacterial pathogenicity^1,14,15^.

Previous structural studies of members of the Fur family of proteins^16–24^ revealed that these transcription factors fold into two distinct domains, including a DNA binding domain (DBD) and a dimerization domain (DD) localized on the N- and C-terminus of the protein, respectively. The DBD is typically composed of a helix-wing-helix domain, while the DD consists of three antiparallel β-strands and two α-helices. Collectively, these structures show the presence of two or three functional metal binding sites. The first structural site, referred to as S1, is located at the C-terminus and is coordinated by four cysteine residues. The second structural site, S3, is also found within the DD and plays a role in stabilizing the dimeric form of the regulator. The third metal binding pocket is the regulatory site (S2) and is composed of residues located in a region referred to as the hinge, which links the DBD and DD.

Classically, the Fur family of metalloregulators was defined as transcriptional repressors coupling metal ion concentrations to gene downregulation^25^. Mechanistically, the metal ion binds to Fur (referred to as the holo-form) to induce a structural reorganization of the DBD, leading to DNA binding and gene repression. However, recent studies revealed that *Campylobacter jejuni*^17^, *Helicobacter pylori*^26–28^ and *Neisseria gonorrhoeae*^29^ Fur proteins both repress and activate gene expression in the absence of regulatory iron (referred to as the apo-form) and also positively regulate gene expression when bound to iron collectively forming four different modes of gene regulation. To better understand the mechanistic basis underlying the extended repertoire of DNA binding by the Fur family of metalloregulators, we undertook structural and biochemical studies of CjFur. In the current study, we show that similar sets of residues are involved in the positive and negative regulation of holo- and apo-regulated genes. Moreover, we demonstrate that metal coordination in the S3 site is important for CjFur DNA binding activity and contributes to processes related to chick ceca colonization by *Campylobacter jejuni*.

## Results

### Mapping the residues involved in apo-Fur regulation

**Figure 1:**
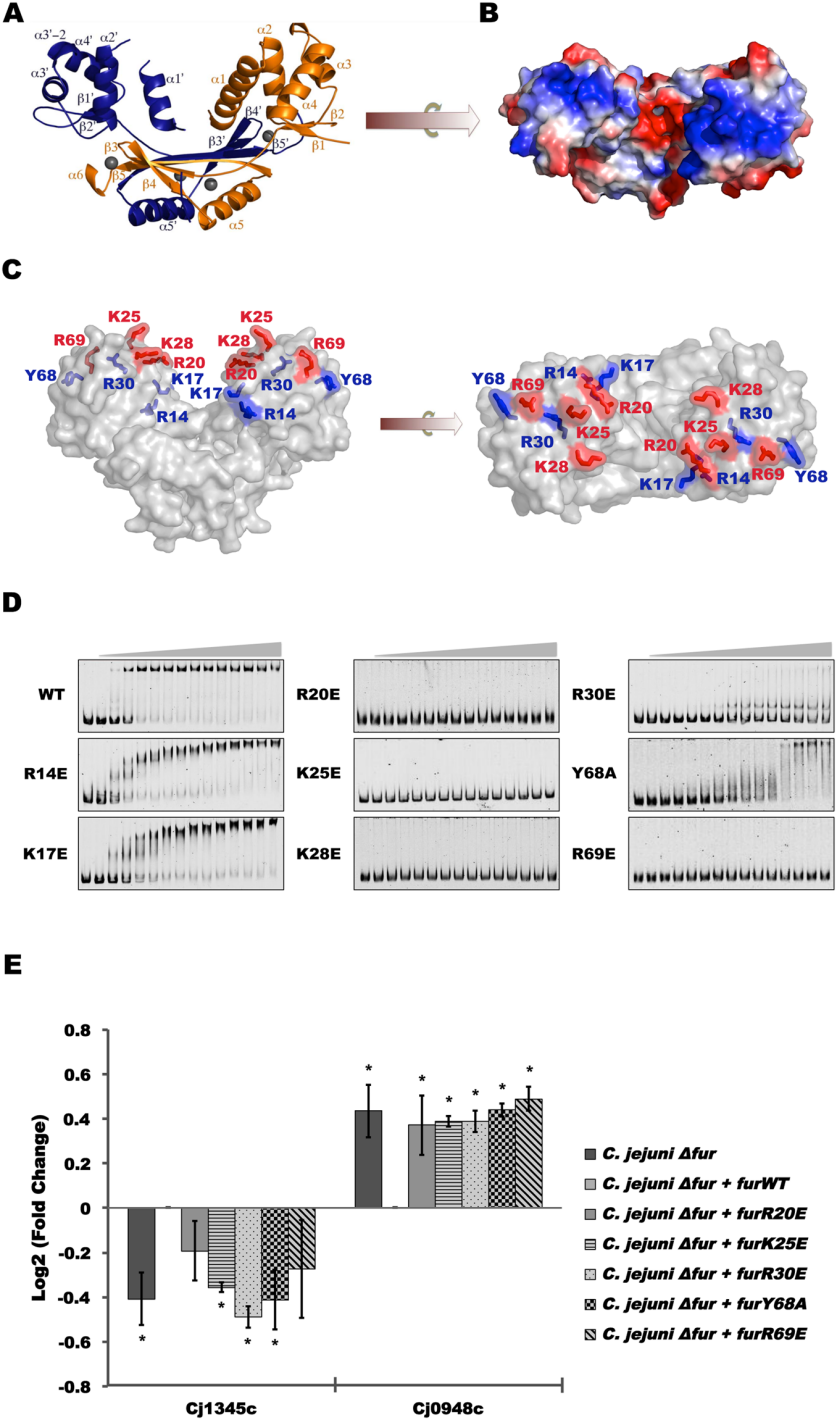
Apo-CjFurZn_2_ structure and regulation. (**A**) CjFur is composed of a N-terminal DNA binding domain and a C-terminal dimerization domain. Protomer A and protomer B are rendered in orange and blue, respectively, and the zinc atoms are represented by gray spheres. Secondary structures are labeled. (**B**) The electrostatic potential surface of apo-CjFur is represented. (**C**) Residues forming the positively charged surface were mutated to glutamic acid or alanine residues. Mutations impairing the DNA binding activity of apo-CjFur are rendered in red, and mutated residues that still bind to DNA are highlighted in blue. (**D**) EMSA of the *cj1345c* promoter region (2 nM) with increasing amounts (0–1000 nM) of CjFurWT, CjFurR14E, CjFurK17E, CjFurR20E, CjFurK25E, CjFurR30E, CjFurY68A and CjFurR69E. (**E**) RT-qPCR analysis of the expression of the Fur-regulated *cj1345c* and *cj0948c* genes in *C. jejuni* Δ*fur*, Δ*fur* + *furWT*, Δ*fur* + *furR20E*, Δ*fur* + *furK25E*, Δ*fur* + *furR30E*, Δ*fur* + *furY68A* and Δ*fur* + *furR69E*. All values are relative to *C. jejuni* Δ*fur* + *furWT* and normalized to *slyD* (endogenous control) and to *fur*.

Gene regulation by CjFur occurs in both the presence and absence of regulatory metals^17^. Based on our initial observations, we believe that our previously published structure of CjFur is devoid of metal in its regulatory site, S2, and still adopts a conformation conducive for binding DNA (Fig. 1A), which exposes a different set of residues on its surface compared to other holo-Fur structures. We posited that alternative regions are involved in binding DNA during holo- and apo-CjFur regulation. To identify these residues, we generated the electrostatic potential map of apo-CjFur^17^. The electrostatic potential shows a positively charged area comprised of basic residues, including R14, K17, K25, K28, R30, and R69 (Fig. 1B,C). To determine the role of this region in apo-CjFur interactions with DNA, we substituted R14, K17, R20, K25, K28, R30 and R69 with glutamic acid. Owing to previous studies^30^ showing the importance of Y68 in the activity of a *E. coli* ferric uptake regulator, we decided to replace Y68 with an alanine residue (Table 1). Each mutant was tested with an electrophoretic mobility shift assay (EMSA) using the promoter region of *cj1345*c (putative periplasmic protein) as the DNA binding element (Fig. 1D). Our results show that the substitutions of R20, K25, K28 and R69 by glutamic acid residues abolish the apo-CjFur binding to the *cj1345c* promoter region. Moreover, the same substitutions of residues K17 and R30 and the replacement of Y68 by alanine significantly reduce apo-CjFur binding to DNA. Overall, these results suggest that R20, K25, K28 and R69 are crucial for apo-CjFur binding to DNA. We then sought to evaluate the changes in the transcriptional activity of these mutants in *Campylobacter jejuni*. We constructed isogenic deletion mutants expressing either wild-type CjFur or the mutants tested above. After confirming that each mutant was expressed at a level similar to the wild-type (Fig. S1), we evaluated the expression of *cj1345c* and *cj0948c* (putative cation efflux protein), two genes known to be activated and repressed by CjFur, respectively, in the absence of iron (Fig. 1E). Consistent with our *in vitro* DNA binding assays, all the mutants failed to activate *cj1345c* expression. Similarly, the same mutants were unable to repress *cj0948c* expression under the same conditions. Overall, these results suggest that R20, K25, K28 and R69 are important for the gene regulatory activity of CjFur. Moreover, our results suggest that similar sets of residues are involved in the activation and repression of gene expression in the absence of iron.

**Table 1:** Summary of the residues important for DNA binding in apo-CjFur and holo-CjFur.

	Regulatory metal ion	Mutations tested	Key residues
Apo-CjFur EMSA	N.A.	R14E, K17E, R20E, K25E, K28E, R30E, Y68A, R69E	R20, K25, K28, R69
Apo-CjFur RT-qPCR (activation)	N.A.	R20E, K25E, R30E, Y68A, R69E	K25, R30, Y68
Apo-CjFur RT-qPCR (repression)	N.A.	R20E, K25E, R30E, Y68A, R69E	R20, K25, R30, Y68, R69
Holo-CjFur EMSA	Mn	R20E, K25E, K28E, R30E, Y68A, R69E	K25, R30, Y68, R69
Holo-CjFur RT-qPCR (repression)	Fe	R20E, K25E, R30E, Y68A, R69E	R20, K25, R30, Y68, R69

N.A. Not applicable.

### Residues involved in holo-Fur regulation

**Figure 2:**
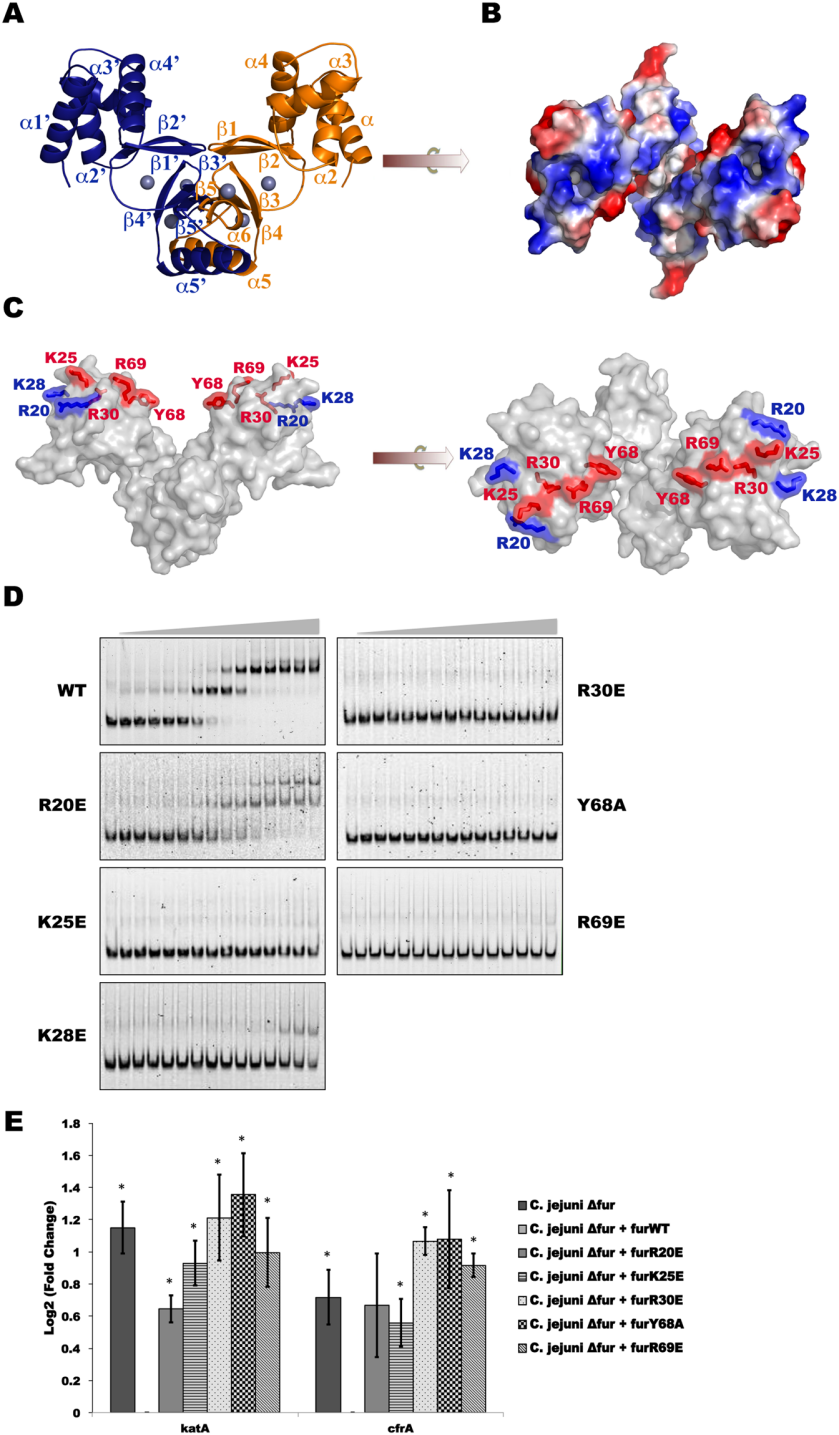
Holo-CjFur structure and regulation. (**A**) Holo-CjFur structure modeled using SWISS_MODEL software. Protomer A and protomer B are rendered in orange and blue, respectively, and the zinc atoms are represented by gray spheres. Secondary structures are labeled. (**B**) The electrostatic potential surface of holo-CjFur is represented. (**C**) Residues forming the positively charged surface were mutated to glutamic acid or alanine. Residues impairing the DNA binding activity of holo-CjFur are rendered in red, and mutated residues that still bind to DNA are represented in blue. (**D**) EMSA of the *katA* promoter region (2 nM) with increasing amounts (0–100 nM) of CjFurWT, CjFurR20E, CjFurK25E, CjFurR30E, CjFurY68A and CjFurR69E. (**E**) RT-qPCR analysis of the expression of the Fur-regulated *katA* and *cfrA* genes in *C. jejuni* Δ*fur*, Δ*fur* + *furWT*, Δ*fur* + *furR20E*, Δ*fur* + *furK25E*, Δ*fur* + *furR30E*, Δ*fur* + *furY68A* and Δ*fur* + *furR69E*. All values are relative to *C. jejuni* Δ*fur* + *furWT* and normalized to *slyD* (endogenous control) and to *fur*.

After determining the residues involved in binding DNA in the absence of regulatory metals, we sought to identify the regions involved in the interaction between holo-CjFur and the *katA* promoter region. We first modeled the holo-CjFur structure using the *Helicobacter pylori* Fur (2XIG)^23^ structure as the template and SWISS_MODEL software^31^. Calculation of the holo-CjFur surface electrostatic potential (Fig. 2A) shows that several positively charged residues (Fig. 2B), including R20, K25, K28, R30, Y68 and R69, cluster in a defined region on the surface of the DBD (Fig. 2C). To determine whether these residues interact with DNA in the presence of regulatory metals, each residue was substituted with a glutamic acid (R20, K25, K28, R30 and R69) or an alanine (Y68). EMSAs show that the mutations of residues K25, R30, Y68 and R69 abolish CjFur binding to the *katA* promoter region (Fig. 2D). The substitution of K28 by a glutamate residue significantly alters DNA binding, and the mutation of R20 reduces, but does not completely abolish, CjFur binding to DNA. Collectively, these results suggest that residues K25, R30, Y68 and R69 are important for binding DNA in the presence of a regulatory metal. The effect of these mutations on the transcriptional activity of Fur in the presence of the regulatory metal ion was examined by measuring the expression levels of holo-CjFur target genes, including *katA* and *cfrA*. As shown in Fig. 2E, all the mutants fail to repress *katA* and *cfrA* gene expression, suggesting that, akin to the residues involved in apo-CjFur gene regulatory activity, a large contingent of basic residues regulates gene expression in the holo-form of CjFur. Moreover, these findings collectively reveal that the same region controls all the gene regulatory mechanisms without relation to the conformational changes observed between the apo- and holo-forms of CjFur.

### Characterization of CjFur S2 and S3 metal binding sites

**Figure 3:**
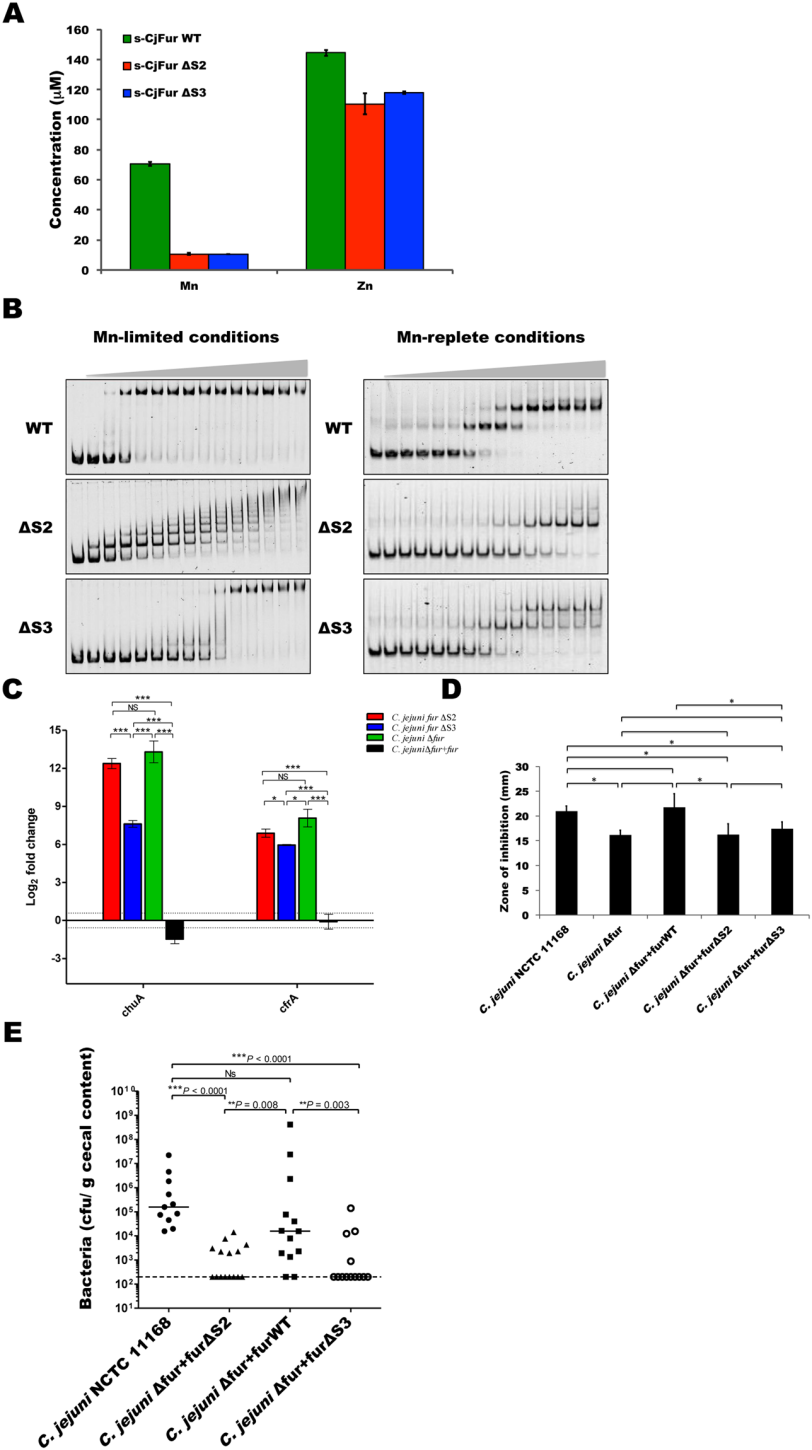
Role of CjFur metal binding sites. (**A**) ICP-MS metal analysis of purified recombinant CjFur, CjFur^ΔS2^ and CjFur^ΔS3^. (**B**) EMSA of the *cj1345c* promoter region (2 nM) and *katA* promoter region (2 nM) in Mn-limited and Mn-replete conditions, respectively, with increasing amounts of CjFur, CjFur^ΔS2^ and CjFur^ΔS3^. (**C**) RT-qPCR analysis of the expression of *chuA* and *cfrA* genes in *C. jejuni* Δ*fur*, Δ*fur* + *fur*, Δ*fur* + *fur*^Δ*S2*^ and Δ*fur* + *fur*^Δ*S3*^ strains. The expression data shown for the *C. jejuni* Δ*fur*, Δ*fur* + *fur* and Δ*fur* + *fur*^Δ*S2*^ were previously reported^51^. (**D**) Growth inhibition assay; 10 μL of 3% H_2_O_2_ was used, and the results were taken 28 hours post H_2_O_2_ exposure. The assays were performed in triplicate using three biological replicates. The statistical significance was determined using the Student’s t-test. P values < 0.05 were considered significant. (**E**) *Campylobacter jejuni* wild type, complemented WT, ΔS2 and ΔS3 colonization levels in the chick cecum. Each data point represents the cfu per gram of cecal content recovered in each chick.

Previous structural studies demonstrated that CjFur adopts a unique three-dimensional structure in which the DBD is rotated by approximately 180° compared to other metalloregulators^17^. In adopting this peculiar conformation, the S3 and the S1 metal binding sites are occupied by zinc atoms, but the residues forming the S2 site are in a conformation non-conducive for binding metals. Based on these observations, it was proposed that the structure represented the conformation of an apo-CjFur. The structure also raised the possibility that the S2 site in CjFur is not important for the activity of the metalloregulator. To test this hypothesis, H43 and H102, two residues predicted to form the S2 binding site, were substituted with alanine residues. The mutant protein, also referred to as CjFur^ΔS2^, was homogeneously purified, and its ability to bind metals was assessed using ICP-MS. As shown in Fig. 3A, consistent with the initial CjFur structure, we detected two molar equivalents of zinc atoms per CjFur protomer and manganese in a 1:1 stoichiometry with the wild-type protein. In contrast, CjFur^ΔS2^ failed to bind manganese. Surprisingly, despite an intact S3 site, analysis of the zinc content revealed that CjFur^ΔS2^ could only metalate one zinc atom per protomer, suggesting that the S2 site is important for the function of the S3 site. We thus investigated whether the S3 site is also important for the metalation of S2 by repeating this experiment with a CjFur mutant (also referred to as CjFur^ΔS3^) in which H99 and H137 were substituted with alanine residues (Fig. 3A). Similar to CjFur^ΔS2^, CjFur^ΔS3^ failed to bind manganese and incorporate a zinc atom in the S3 site. This suggests that both the S2 and S3 sites interact with each other and that these interactions are important for binding manganese/zinc at these locations.

The binding of metals in the regulatory site of Fur proteins is important for the DNA binding activity. Given that the S2 and S3 sites are important for the binding of manganese and zinc, we posited that both sites are important for DNA binding activity. To test this hypothesis, we performed binding reactions with increasing concentrations of wild-type CjFur, CjFur^ΔS2^ or CjFur^ΔS3^ and a DNA probe corresponding to the promoter region upstream of the *cj1345c* operon, a region known to be regulated by CjFur in apo-conditions. As shown in Fig. 3B, in binding conditions devoid of metals, wild-type CjFur binds to DNA with high affinity, while CjFur^ΔS2^ generates several distinguishable CjFur^ΔS2^/DNA complexes. Analogously, mutation of the S3 metal binding site negatively affects CjFur DNA binding activity. To measure the differences in the impact of impairing the S2 or S3 metal binding site between the apo- and holo-conditions, we repeated the binding assays in the presence of an excess of metals and a DNA fragment corresponding to the promoter of the holo-regulated gene *katA*. Comparative analysis between the binding assays performed in the holo- and apo-conditions with the wild-type proteins reveals that in contrast to the apo-conditions, wild-type CjFur forms two distinguishable binding species. In contrast, in the range of concentrations tested, CjFur^ΔS2^ fails to generate two different DNA-bound CjFur complexes and shows weaker binding to *katA* (Fig. 3B**)**. Similarly, CjFur^ΔS3^ binds with less affinity to DNA; however, the impact of its mutation is less deleterious in the holo-conditions compared to the binding reactions performed in the apo-conditions. Overall, these observations suggest that both metal binding sites play a role in properly aligning the CjFur DBD in an orientation conducive for binding DNA.

To assess the potential role of CjFur S2 and S3 metal binding sites *in vivo*, we complemented the CjFur-deleted strain (referred to as Δ*fur*) using CjFur wild-type (Δ*fur* + *fur)*, *CjFur*^Δ*S2*^ (Δ*fur* + *fur*^ΔS2^*)* or *CjFur*^Δ*S3*^ (Δ*fur* + *fur*^ΔS3^*)* genes. Consistent with the *in vitro* binding assays, stable expression of CjFur^ΔS2^ and CjFur^ΔS3^ in a Δ*fur* background strain failed to maintain the repression of two holo-regulated genes, *chuA* and *cfrA* (Fig. 3C), while complementation with the wild-type *fur* gene restored the repression of these genes. Consistent with the ability of CjFur to confer resistance to reactive oxygen species (ROS)^32^, the Δ*fur* background strain mutant exhibited increased sensitivity toward H_2_O_2_ relative to the NCTC strain or the Δ*fur* + *fur* strain. In contrast to the Δ*fur* strain, both the Δ*fur* + *fur*^ΔS2^ and Δ*fur* + *fur*^ΔS3^ strains failed to show H_2_O_2_ resistance (Fig. 3D). These results indicate that both the CjFur S2 and S3 sites play an important function in resistance to hydrogen peroxide in *C. jejuni*.

To determine whether the CjFur S2 and S3 regulatory sites are important in the colonization of chick ceca, three-day-old chicks were inoculated with either wild-type *C. jejuni* NCTC11168, Δ*fur* + *fur*, Δ*fur* + *fur*^ΔS2^, or Δ*fur* + *fur*^ΔS3^ *strains*. As shown in Fig. 3E, both *C. jejuni* NCTC11168 and the Δ*fur* + *fur* strains colonized the ceca at a median of approximately 5 × 10^4^ CFU/g. Conversely, both the Δ*fur* + *fur*^ΔS2^ and the Δ*fur* + *fur*^ΔS3^ *strains* failed to colonize the ceca relative to the wild-type, with both showing an average reduction of more than 99% in colonization. Overall, these results indicate an important role for the CjFur S2 and S3 sites in chick colonization.

### Crystal structure of CjFurZn

**Figure 4:**
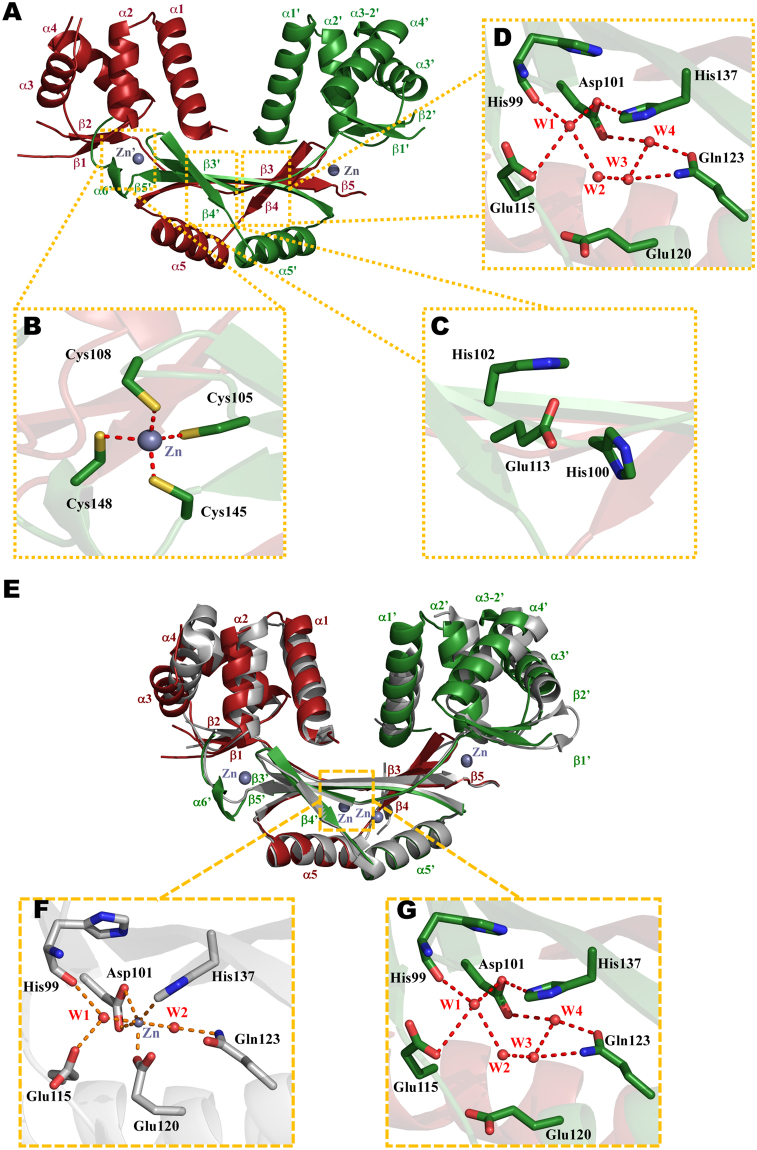
CjFurZn mimics CjFurΔS3. (**A**) Cartoon representation of CjFurZn in which one protomer is highlighted in green and the other subunit is colored in red. The zinc atoms are represented as gray spheres. Zoomed view on the S1 (**B**), S2 (**C**) and S3 (**D**) metal binding sites in which oxygen and nitrogen atoms are rendered in red and blue, respectively. (**E**) Structural alignment of CjFurZn and CjFurZn_2_ structures. CjFurZn is represented as in A, while CjFurZn_2_ is in gray. Zoomed view on the S3 site of CjFurZn_2_ (**F**) and CjFurZn (**G**). Carbon atoms of CjFurZn_2_ are rendered in gray, while CjFurZn atoms are colored as in panel B.

To further understand the role of the S3 site in CjFur DNA binding activity, we carried out crystallization trials to solve the structure of the wild-type transcription factor devoid of metal in its S3 site. After screening several crystals obtained in various conditions, we obtained a unique crystal form in a condition containing PEG3350 as a precipitant and Mg-Formate as a salt. The crystal structure of the new CjFur was solved by molecular replacement using the CjFur DBD and DD as search models. The model was built and refined to a final R_work_/R_free_ ratio of 18.3%/22.9% at a 1.8 Å resolution. The bond lengths in the final model have a root mean square deviation (RMSD) value of 0.01 Å, and the angles fall within favored and allowed regions (98.9% favored and 0.8% allowed) of the Ramachandran plot (Table 2). Only K97 in one of the monomers was modeled with bond angles not allowed by Ramachandran’s rules and steric repulsion constraints. The structure shows that the protein assembles into a biological dimer (Fig. 4A) in which each monomer folds into two domains, the DBD and the DD, reminiscent of other ferric uptake regulators. The DBD contains 5 α-helices (α1, α2, α3, α3-2 and α4) and β-strands β1 and β2. The DD contains three β-strands (β3–β5) intersected by one α-helix (α5). The α1-helix of the CjFur DBD faces the inner portion of the V-cleft and is maintained in an orientation conducive for binding DNA. Close inspection of the metal binding sites revealed that one of the two metal atoms characterized in the previous apo-CjFur structure could be detected in the new apo-CjFur. The modeled zinc atom is tetra-coordinated by C105, C108, C145 and C148 in the S1 metal binding site. The length of all four coordination bonds is approximately 2.3 Å (Fig. 4B), a value consistent with measurements of the S1 site in other metalloregulators^17,33^. Despite adopting a V-shape structure, no metals could be modeled in the S2 sites (Fig. 4C). Intriguingly, akin to the S2 site, no metals could be detected in the S3 site (Fig. 4D). Instead, four water molecules establish several hydrogen bonds with residues forming the S3 sites. In reference to the previous crystal structure in which the S1 and S3 sites were metalated, which will be referred to as CjFurZn_2_, the new crystal structure will be referred to as CjFurZn.

**Table 2:** Data collection, phasing and refinement statistics for the crystal structure of CjFurZn.

**Data collection**	CjFurZn
Space group	P2_1_2_1_2_1_
Cell dimensions	
a, b, c (Å)	35.74, 84.36, 123.63
Resolution	69.68–1.81
Rsym	6.3 (48.3)^!^
I/σI	5.3 (2.9)
Completeness (%)	100 (100)
Redundancy	3.4 (2.1)
**Refinement**	
Resolution	34.65–1.81
Reflections	34641
R_work_/R_free_	18.6/23.1
No. atoms	
Protein	2369
Zn^2^^+^	2
Water	398
B-factors (Å^2^)	
Protein	25.8
Zn^2^^+^	13.7
Water	37.6
R.m.s. deviations	
Bond lengths (Å)	0.016
Bond angles (°)	1.33
Molprobity score	1.59
Ramachandran favored (%)*	98.9

^!^Highest resolution shell.

*There are no Ramachandran outliers.

Comparative analyses between CjFurZn and CjFurZn_2_ reveal that both metalloregulators align relatively well with respect to their dimerization domains. However, close inspection of the structural alignment of their DBDs reveal several structural differences (Fig. 4E). The DBD of CjFurZn protomer A (Fig. 4E) undergoes a clockwise rotation of ~5°, shifting the C-terminus of its α1-helix inwardly by ~5 Å. The rotation also displaces the α4-helix C-terminus by an equivalent distance and moves the N-terminus of the α3-helix by ~3 Å outward of the V-shaped cleft. The DBD of CjFurZn protomer B does not undergo a rotation, but instead, its α3- and α4-helices are displaced toward the inner portion of the V-shaped cleft. Altogether, these observations show that the DBD can undergo asymmetric reorganization when the S3 site is not metalated.

After observing notable structural reorganization of the CjFurZn DBD, we posited that the orientation of residues forming the S3 site would differ between CjFurZn and CjFurZn_2_. In CjFurZn_2_, the Zn^2^^+^ ion is hexacoordinated by residues Asp101, Glu120, His137 and two water molecules (Fig. 4F). One water molecule makes a short hydrogen bond with the backbone carbonyl of His99 and forms an electrostatic interaction with the zinc atom. The other water molecule bridges an interaction between the side chain’s amide of Gln123 and the zinc atom (Fig. 4F). In contrast to CjFurZn_2_, two additional water molecules occupy CjFurZn S3 site (Fig. 4G). One of these water molecules makes a water-mediated hydrogen bond with Glu115 carboxylate, while the other water molecule exhibits a direct electrostatic interaction with the Gln128 side chain (Fig. 4G). Moreover, comparative analysis of the CjFurZn and CjFurZn_2_ S3 sites shows that the rotation of Glu120 side chain places its carboxylate group away from the S3 site that likely enables the binding of the two additional water molecules. The side chain of Glu115 also rotates around its C_β_ to optimize the hydrogen bonding with one of the water molecules. Altogether, these results show that while the loss of metal binding in the S3 site can be compensated by water molecules, it causes a distortion in the DBD that negatively affects DNA binding by CjFur.

## Discussion

A recent study showed^17^ that CjFur protein recognizes several DNA-binding motifs depending on the regulatory mode. Consequently, novel consensus sequences controlling the holo-Fur activated and holo-Fur repressed gene regulatory programs were identified. However, the biochemical basis underlying this expanded repertoire of DNA sequences remained unknown. To better understand the mechanistic basis underlying the binding of CjFur to different target promoters, we performed DNA binding assays in holo- and apo-conditions. To perform our binding studies, we used a DNA element corresponding to the promoter region of *cj1345c*, as this gene was found to be apo-regulated using microarray and ChIP-Chip analysis^17,34^. Moreover, this gene was shown to be differentially expressed in a Δ*fur* mutant under iron-limited conditions. DNA binding assays performed in manganese-limited conditions with increasing amount of apo-CjFur showed the presence of one main shifted species migrating at a relative migration distance (Rf) of 0.07. However, upon substitution of R30 with a glutamic acid, an intermediary shifted species migrating at a Rf of 0.31 was observed. While the stoichiometry of binding of apo-CjFur to DNA is still unknown, our data suggest that wild-type CjFur, in apo-conditions, forms oligomers on DNA and that R30 is important for this multimerization. Interestingly, the Protein Interfaces, Surfaces and Assemblies (PISA) analysis revealed that the homologous arginine residue in PaFur, R18, is important for its tetramerization^35^, suggesting that this residue could also be involved in the multimerization of CjFur on DNA (Table 2). Similarly, recent structural studies revealed that the binding of EcZur to DNA is achieved by cooperative binding of two adjacent EcZur dimers. Such cooperativity arises from two salt bridges between D49 and R52 residues that connect two adjacent independent dimers^36^. It is therefore tempting to speculate that apo-CjFur R30 and E31 play an analogous role in the interaction of adjacent CjFur dimers when bound to DNA.

In manganese-replete conditions, small increments in the protein concentration enabled us to observe two shifted species in EMSAs. Concentrations ranging from 0.1 to 4.0 nM yielded a CjFur-DNA complex migrating at an Rf of 0.37, while increasing the protein concentration from 8 to 100 nM generated a nucleo-protein complex with a larger apparent molecular weight, migrating at an Rf of 0.24, a binding profile reminiscent of those in previous binding studies of EcZur with DNA^36^. In these studies, an intermediary species was observed and assigned as an EcZur-DNA complex with a 1:1 stoichiometry, while the higher molecular weight species was observed to be composed of two Zur dimers and one DNA molecule. These binding studies are in agreement with the binding model in which the Fur box contains recognition sites for two Fur dimers binding to opposite faces of the DNA helix^37^. Moreover, the presence of two shifted species is consistent with the crystal structures of *Magnetospirillum gryphiswaldense* Fur and *Escherichia coli* Zur in complex with DNA. Both studies revealed the presence of two dimers on the promoter region, with one protomer of each dimer binding one half of the Fur box^36,38^. In line with these findings, we conclude that the intermediary shifted species represents one holo-CjFur dimer in complex with the 60 bp *katA* promoter fragment, while the higher molecular weight species is formed by two dimers of holo-Fur bound to the same DNA element. However, DNA binding assays do not always result in the presence of two shifted species as recent studies in PaFur and *Francisella tularensis* Fur showed that in these proteins a single Fur dimer binds their target Fur box^35^. Moreover, the ability of the Fur protein to form higher molecular weight complexes depends on the DNA target. For example, holo-MgFur can bind to both the *feoAB1* operator and the *P. aeruginosa* Fur box, but the binding mode is different between each one^38^. While there was only one shifted species for both DNA fragments, the MgFur- *feoAB1* complex had a higher mobility compared to the MgFur-PaFur-box complex, suggesting divergent binding stoichiometries. These results were further supported by the crystal structures of the MgFur-*feoAB1* operator and MgFur-PaFur box complexes, which showed that one Fur dimer binds to double-stranded DNA in the holo-MgFur - *feoAB1* operator structure, while the structure of the holo-MgFur- *P. aeruginosa* Fur box complex contained two Fur dimers^38^ bound to one DNA duplex. Altogether, these studies suggest that the DNA binding mechanisms of Fur proteins are modulated by both the presence of the regulatory metal ion and the DNA sequence.

To gain functional information about *C. jejuni* Fur regulation and potentially identify unique or shared apo- and holo-specific DNA binding residues, we performed a detailed site-specific mutagenesis study predominantly targeting positively charged residues located in the DNA binding domain of CjFur. The same mutants were assayed in both in holo- and apo-conditions. In holo-conditions, substitution of residues K25, R30, Y68 and R69 blunted CjFur interaction with DNA and its ability to repress gene expression. These observations are consistent with the crystal structure of the MgFur-DNA complex showing that K15 (corresponding to K25 in CjFur) recognizes DNA through shape readout, while Y56 and R57 (corresponding to Y68 and R69 in CjFur) recognize the substrate through base readout^38^ (Table 3). In apo-conditions, substitution of R20, K25, K28 and R69 yielded an inactive CjFur *in vitro* and *in vivo*. While a crystal structure of an apo-CjFur-DNA complex still remains to be solved, our results suggest that these positively charged residues make direct contacts with DNA; however, the surface of interaction likely differs from the holo-CjFur. This idea is supported by our observation that R20E and K28E mutants maintain noticeable binding activity in the presence of regulatory metals, while in apo-condition, these substitutions result in a complete of loss of binding. Collectively, these findings suggest that apo-CjFur binds DNA differently than holo-CjFur.

**Table 3:** Summary of DNA binding residues identified in this study and those involved in contacting DNA identified in the crystal structures of Fur/DNA and Zur/DNA complexes.

		Residues	Protein^(reference)^
This study	Residues identified in this study	R20, K25, R30, Y68, R69	CjFur^(this study)^
	DNA binding residues interacting with DNA bases	K15 (K25), Y56 (Y68), R57 (R69)	MgFur^52^
		Y45, R65 (R69)	EcZur^53^
Previous studies	DNA binding residues interacting with phosphodiester backbone	V16, T17, Q19, R20 (R30), S51, T54, R77, A78	MgFur^52^
		R23 (K25), T25, Q27, R28 (R30), Y64 (Y68)	EcZur^53^

Residues in brackets correspond to CjFur residues identified in this study.

### The interplay of CjFur S2 and S3 regulatory sites

**Figure 5:**
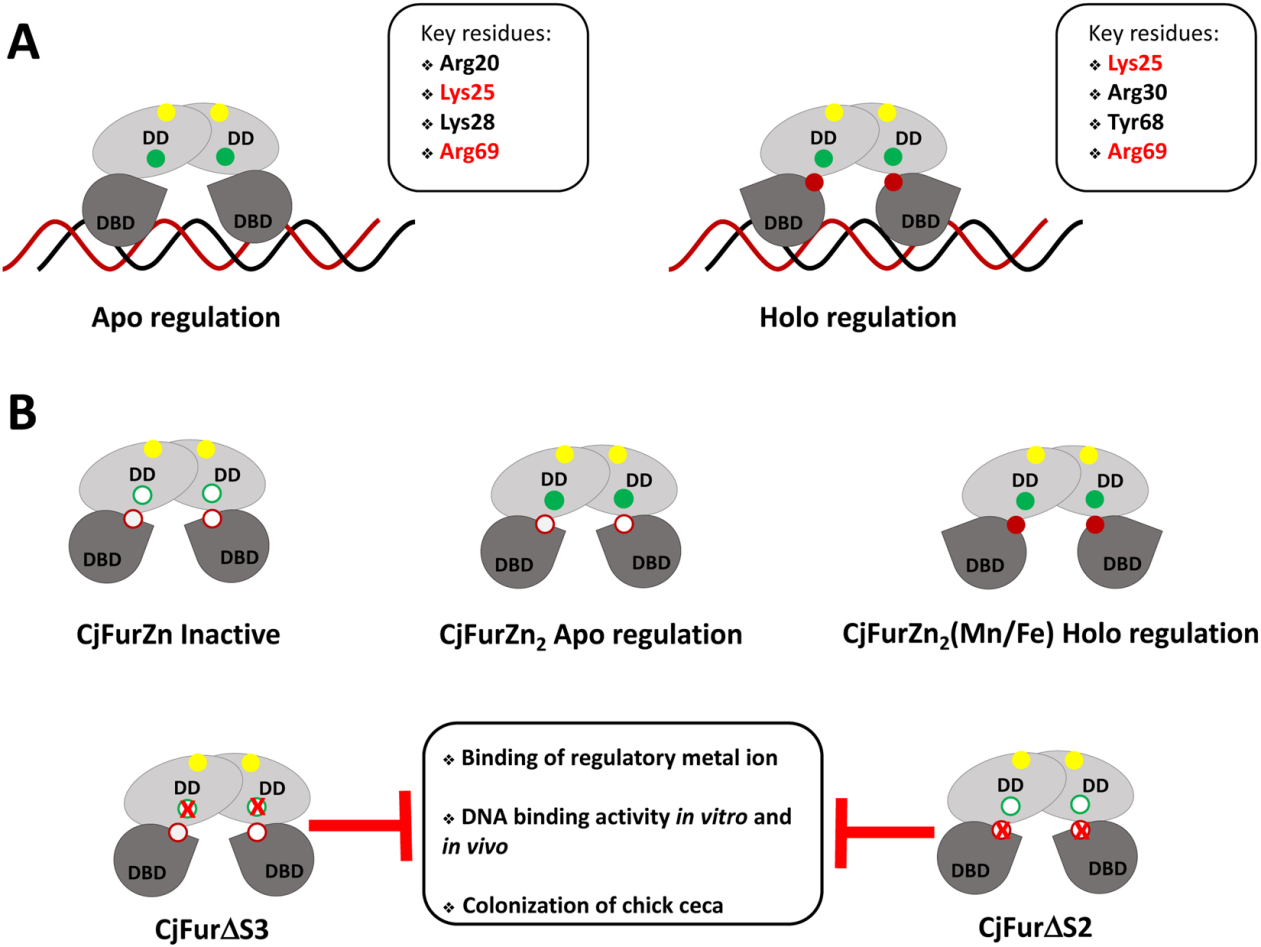
The interplay between the S2 and S3 sites maximize CjFur DNA binding activity. (**A**) Schematic representation of apo-CjFur and holo-CjFur regulation. Key residues important for the gene regulatory activity of CjFur are listed and residues highlighted in red represent residues involved in both apo and holo regulation. Yellow, red and green circles represent S1, S2 and S3 metal binding sites, respectively. (**B**) Schematic representation of different metalation states of CjFur presented in this study. Filled and empty circles indicate metalated and unoccupied metal binding sites, respectively. The red X represent mutated metal binding sites. Mutations of S2 and S3 metal sites result in loss of metal binding in both S2 and S3 sites, decreased DNA binding activity *in vitro* and in *C. jejuni* and decreased ability to colonize chick ceca.

Initial structural studies of CjFur highlighted the ability of the metalloregulator to maintain a conformation conducive for binding DNA in the absence of metal at the S2 regulatory site, raising the intriguing possibility that the S2 site may not be necessary for the activity of CjFur. To address this question, we substituted residues forming the S2 site. Consistent with previous studies, these substitutions rendered the metalloregulator inactive, blunting both its DNA binding activity *in vitro* and its gene regulatory activity *in vivo*. Moreover, ICP-MS analysis revealed that CjFur can simultaneously metalate 1 manganese atom and 2 zinc ions, demonstrating that, akin to other ferric uptake regulators, CjFur holo-regulates gene expression and that its S2 site is important for such activity. Surprisingly, upon mutation of residues forming the S3 site, CjFur lost its ability to bind manganese, suggesting that a network of interactions mediate an interplay between the S2 and S3 sites. Combined with the crystal structure of CjFurZn, these observations suggest that the S3 site plays two important functions in CjFur biology, in contributing to S2 metalation and in correctly aligning the DNA binding domain of CjFur for the control of apo-regulated genes. Previous studies documented a similar interplay in *Bacillus subtilis* and *Helicobacter pylori* Fur showing that mutation of the S3 site impacts the incorporation of metals in both metal binding sites^39,40^. However, we note that BsFur mutants in S2/S3 were unaffected in DNA binding under saturating manganese conditions while CjFur^ΔS2^ and CjFur^ΔS3^ mutants showed defects even under 500 molar excess of manganese. HpFur^ΔS2^ was completely unable to bind its cognate promoter, with HpFur^ΔS3^ only slightly affected. In contrast, CjFur^ΔS2^ was able to bind to its target under holo-conditions but was unable to form a higher molecular weight complex indicative of full binding as seen in the WT CjFur. CjFur^ΔS3^ showed a slight reduction in holo-binding as seen in HpFur^ΔS3^ but showed a much more obvious binding defect under apo-conditions. Unfortunately, there have been no studies looking at HpFur S2/S3 mutant binding under apo-conditions as reported for CjFur herein. It would be interesting to see if the HpFur S2/S3 site mutants have similar impact on apo-HpFur binding as we observed for apo-CjFur. In addition, zinc binding to BsZur has also demonstrated an interplay between the S2/S3 sites and their respective affinities for zinc ions^41^. Maximal BsZur binding to its DNA binding consensus sequence was only achieved once both S2/S3 sites were metalated and there was negative cooperativity between metalation of the S2 and S3 sites. In contrast with these results, and those with BsFur, HpFur as well, our results demonstrate that the presence of both S2/S3 sites are required for proper metalation of holo-CjFur (Fig. 5).

In addition of blunting metal incorporation, mutation of the S2 and S3 metal binding sites also impairs CjFur ability to form higher molecular weight complexes. As shown in *H. pylori*, iron binding impacts the ability of Fur to form higher-order structures^42^ and mutation of residues involved in the coordination of metal ions in S2 and S3 sites in *Helicobacter pylori* namely residues His96 (S3 site) and Glu110 (S2 site) resulted in the reduced ability to form higher-order structures^42^. Collectively, these results suggest that these mutations induce conformational changes that position residues important for the interaction of different CjFur dimers on DNA element in one or multiple orientations non-conducive for protein oligomerization.

In conclusion, considering that (1) CjFur is essential for gut colonization and *Campylobacter jejuni* life cycle, (2) no Fur homologues are found in eukaryotes and 3) the S3 site is essential for the activity of CjFur in terms of contributing to both the metalation of S2 and the correct alignment of the CjFur DNA binding domain, our results collectively highlight a potentially novel approach to block CjFur function in developing antimicrobial agents targeting the S3 metal binding sites.

## Material and Methods

### CjFur purification

To obtain the metal bound form of CjFur, its cDNA was cloned into a homemade vector, enabling the expression of the metalloregulator as an N-terminal TEV-cleavable Strep tag. CjFur was overexpressed in *E. coli* Rosetta cells for 16 hours at 18 °C with 100 μM IPTG. Following overexpression, cells were centrifuged and harvested in 50 mM Tris pH 7.0, 100 mM NaCl and 5 mM β-mercaptoethanol (Bme). CjFur was purified on Strep-TACTIN beads as previously described^17,43^. Following elution and TEV cleavage, CjFur was concentrated and further purified by size-exclusion chromatography using a Superdex 75 pre-equilibrated with 20 mM Tris pH 7.0, 250 mM NaCl and 5 mM Bme. The protein was concentrated and stored at −80 °C.

### Metalation of CjFur

Purified CjFur was incubated with excess MnCl_2_ (3:1 metal to protein molar ratio), which was gradually added to the protein, with 5 minutes of incubation on ice between each addition. To remove the excess of free metal ions, a second size exclusion chromatography was performed in 20 mM Tris pH 7.0, 250 mM NaCl and 5 mM Bme. After this second gel filtration, purified holo-CjFur was concentrated and used for electrophoretic mobility shift assays.

### Inductively Coupled Plasma Mass Spectrometry Metal Analysis (ICP-MS)

The metal content of purified CjFur proteins was determined by inductively coupled plasma mass spectrometry (ICP-MS) at the Quantitative Bioelemental Imaging Center, Northwestern University, using a Thermo Fisher X Series II ICP-MS system. Using two serial dilutions (1/20 and 1/100) of purified CjFur and recording 3 spectra per dilution, a total of six MS spectra per protein sample were recorded and used for metal content calculations.

### Electrophoretic Mobility Shift Assays (EMSA)

To perform EMSAs mirroring the holo-form of the transcription factor, forward (CjkatA60_For_1588) and reverse (CjkatA60_Rev_1589) (Table S2) Cy5-labeled primers (Eurofins MWG operon) corresponding to a 60 bp DNA fragment of the *katA* promoter region were annealed by incubation at 95 °C for 15 minutes and slowly cooled to room temperature. For the gel shift assays, 2 nM Cy5-labeled DNA fragment was incubated with increasing amounts (0–100 nM) of purified recombinant holo-CjFur protein for 45 minutes on ice in binding buffer (20 mM Bis-Tris borate, pH 7.4, 50 mM KCl, 3 mM MgCl_2_, 5% glycerol, 0.1% Triton X-100, 0.1 μg poly dI-dC non-specific DNA competitor, and 50 μM MnCl_2_). Samples were separated on a 6% non-denaturing polyacrylamide gel (19:1) for 1 hour at 100 V and 4 °C. Gels were freshly prepared with 100 mM Bis-Tris borate, pH 7.4, and 100 μM MnCl_2_ and prerun for 30 minutes at 150 V and 4 °C.

To perform EMSAs in conditions mirroring the apo-form of CjFur, the promoter region upstream of the *cj1345c* operon (200 bp) was PCR amplified using the *Campylobacter jejuni* NCTC chromosomal DNA as a template and the *cj1345c*_For_1692/*cj1345c*_Rev_1693 (Table S2) (Eurofins MWG Operon) pair of primers with the forward primer labeled with Cy5. For the gel shift, approximately 2 nM Cy5-labeled DNA fragment was incubated on ice with increasing amounts (0–1000 nM) of purified recombinant apo-CjFur protein in binding buffer (20 mM Bis-Tris borate, pH 7.4, 50 mM KCl, 3 mM MgCl_2_, 5% glycerol, 0.1% Triton X-100 and 0.1 μg of poly dI-dC) for 45 minutes. Samples were then loaded on 5% nondenaturing polyacrylamide gel (19:1) and electrophoresed for 1 hour at 100 V and 4 °C. Gels were freshly prepared with 100 mM Bis-Tris borate, pH 7.4, and prerun for 30 minutes at 150 V and 4 °C.

### Construction of complemented mutant *Campylobacter jejuni* NCTC11168 strains

The FurR20E, FurR30, FurY68A and FurR69E gene regions were amplified from the corresponding pStrepFur constructs. Amplified PCR products were then cloned into the pRRK vector^44^ using the In-Fusion-Dry-Down PCR cloning kit. All the mutants were then sequenced to confirm the absence of PCR-induced errors in the inserts. The *Campylobacter jejuni* NCTC11168 Δ*fur* mutants were then transformed with these Fur mutant constructs, and positive colonies were selected on MH agar plates supplemented with 20 μg/mL chloramphenicol and 10 μg/mL kanamycin. The insertion of different mutant genes was confirmed by PCR and sequencing.

### RT-qPCR analysis

Cells were grown under microaerophilic conditions to midlog phase in MEM-α medium supplemented with 10 mM sodium pyruvate with or without 40 μM FeSO_4_. Ten percent cold stop solution (10% buffer-saturated phenol in absolute ethanol) was added to cell cultures, and the cells were harvested by centrifugation at 4000 RPM for 10 minutes. Cell pellets were resuspended in TE buffer (10 mM Tris-HCl, pH 8.0, and 1 mM EDTA), and the RNA was extracted using the hot phenol-chloroform method^45^. RNA precipitation was performed by an overnight incubation in absolute ethanol at −80 °C. RNA pellets were subsequently washed four times with 80% cold ethanol and were resuspended in RNase-free water. RNA was then treated with RNase-free DNase I (Invitrogen) to remove contaminating genomic DNA and was further purified using an RNeasy Mini Kit (Qiagen). The absence of contaminating genomic DNA was confirmed by PCR, and the quality of RNA was assessed on agarose gel. Reverse transcription was carried out using the QuantiTect Reverse Transcription Kit (Qiagen). RT-qPCR was performed with the MX3000P platform (Stratagene) using the Syber Green quantification method and ROX normalization. The relative expression levels of *katA* and *cfrA* as well as *cj1345c* and *cj0948c*, two holo-Fur-regulated and apo-Fur-regulated genes, respectively, were analyzed by RT-qPCR. The relative expression level of each gene was normalized to *slyD* (endogenous control) and to *fur*. The primers set used for *katA*, *cfrA*, *cj1345c* and *cj0948c* are listed in Table S2. The comparative CT (ΔΔCT) method was used to determine the relative gene expression. All experiments were performed in triplicate, and statistical significance was determined using the Student’s t-test. P values < 0.05 were considered significant.

### Disc inhibition assay

*Campylobacter jejuni* NCTC11168, Δ*fur* deletion mutant and complemented fur, fur^ΔS2^ and fur^ΔS3^ strains were grown on MH agar plates supplemented with required antibiotics (chloramphenicol, kanamycin or both) for three days under microaerophilic conditions. Several colonies from each strain were cultured overnight in MH biphasic flasks. The overnight cell cultures were diluted in MH broth to an optical density at 600 nm of 1.0. For each strain, 4 mL of this diluted bacterial suspension was added to 90 mL of melted MH agar (cooled to approximately 50 °C), and the mixture was poured in equal volumes into three Petri dishes. After solidification, paper discs (6 mm diameter) were placed on the surface of the agar, and 10 μL of 3% hydrogen peroxide (H_2_O_2_) was added to each paper disc. MH agar plates were subsequently incubated under microaerophilic conditions for 20 hours, and the diameter of growth inhibition was measured in millimeters for each strain. All experiments were performed in triplicate, and statistical significance was determined using the Student’s t-test. P values < 0.05 were considered significant.

### Chick colonization assay

The chick colonization assay was performed as described previously^34^. Briefly, strains corresponding to *Campylobacter jejuni* NCTC11168 or strains harboring a deletion in the *fur* gene (Δ*fur)* and complemented with either wild-type *fur* (Δ*fur* + *fur*), Δ*fur* + *fur*^Δ*S2*^ and Δ*fur* + *fur*^Δ*S3*^ were grown to mid-log phase in biphasic flasks under microaerophilic conditions at 37 °C. Bacterial suspensions were then diluted in fresh MH broth to an optical density at 600 nm of 0.13, which corresponds to approximately 10^8^ CFU/mL.Water and food were withheld for 2 hours prior to inoculation. Three-day-old chicks were inoculated orally with 300 μL of the diluted bacterial suspension. Each bacterial suspension was serially diluted and plated on MH-agar plates to confirm that all the chicks received approximately the same number of viable *C. jejuni*. The chicks were euthanized seven days post inoculation, and their ceca were collected and weighed. The cecal contents were extracted and homogenized in MH broth. The cecal contents were then serially diluted and plated onto selective Karmali agar (Oxoid) supplemented with chloramphenicol and kanamycin for the complemented strains. The Karmali plates were incubated under microaerophilic conditions for 48 hours at 42 °C, and the resulting colonies were counted and expressed as CFU per gram of ceca. The data were statistically analyzed with a nonparametric Mann-Whitney rank sum test. P values less than 0.05 were considered statistically significant.

### Crystal structure of the CjFurZn structure

The crystals of CjFur devoid of metals in the S2 and S3 regulatory sites were obtained as follows. Following gel filtration and concentration, CjFur (7.5 μg/μL) was immediately combined, in a 1:1 ratio, with a mother liquor composed of 0.1 M Bis-Tris pH 5.5, 0.25 M MgFormate, and 25% PEG3350. Crystals were harvested and soaked in the mother liquor supplemented with 7.5% glycerol. A full dataset was collected at the 17-ID beamline at Argonne National Laboratory’s Advanced Photon Source and indexed using HKL-2000 (HKL Research). The structure of CjFurZn was solved by molecular replacement using the CjFur DBD and DD (PDB: 4ETS) as search models and the program Phaser from the PHENIX package^46^. The model was refined using the BUSTER software^47^, and manual modifications to the model were done in Coot^48^. The quality of the model was assessed using Molprobity^49^ and RAMPAGE^50^. Coordinates were deposited under the accession number 6D57.

## Electronic supplementary material

Supplementary Information

## References

[1] Troxell B, Hassan HM (2013). Transcriptional regulation by Ferric Uptake Regulator (Fur) in pathogenic bacteria. Front Cell Infect Microbiol.

[2] Lee JW, Helmann JD (2007). Functional specialization within the Fur family of metalloregulators. Biometals.

[3] Giedroc, D. P. & Arunkumar, A. I. Metal sensor proteins: nature's metalloregulated allosteric switches. *Dalton Trans*, 3107–3120, 10.1039/b706769k (2007). 10.1039/b706769k 17637984

[4] Fillat MF (2014). The FUR (ferric uptake regulator) superfamily: diversity and versatility of key transcriptional regulators. Arch Biochem Biophys.

[5] Ahn BE (2006). Nur, a nickel-responsive regulator of the Fur family, regulates superoxide dismutases and nickel transport in Streptomyces coelicolor. Molecular Microbiology.

[6] Bsat N, Herbig A, Casillas-Martinez L, Setlow P, Helmann JD (1998). Bacillus subtilis contains multiple Fur homologues: Identification of the iron uptake (Fur) and peroxide regulon (PerR) repressors. Molecular Microbiology.

[7] Díaz-Mireles E (2004). The Fur-like protein Mur of Rhizobium leguminosarum is a Mn2+ - responsive transcriptional regulator. Microbiology.

[8] Gaballa A, Helmann JD (1998). Identification of a zinc-specific metalloregulatory protein, Zur, controlling zinc transport operons in Bacillus subtilis. Journal of Bacteriology.

[9] Hamza I, Chauhan S, Hassett R, O'Brian MR (1998). The bacterial irr protein is required for coordination of heme biosynthesis with iron availability. Journal of Biological Chemistry.

[10] Patzer SI, Hantke K (1998). The ZnuABC high-affinity zinc uptake system and its regulator Zur in Escherichia coli. Molecular Microbiology.

[11] Platero R, De Lorenzo V, Garat B, Fabiano E (2007). Sinorhizobium meliloti fur-like (Mur) protein binds a fur box-like sequence present in the mntA promoter in a manganese-responsive manner. Applied and Environmental Microbiology.

[12] Butcher J, Handley RA, van Vliet AH, Stintzi A (2015). Refined analysis of the Campylobacter jejuni iron-dependent/independent Fur- and PerR-transcriptomes. BMC Genomics.

[13] Palyada K, Threadgill D, Stintzi A (2004). Iron acquisition and regulation in Campylobacter jejuni. J Bacteriol.

[14] Troxell B (2011). Fur negatively regulates hns and is required for the expression of HilA and virulence in Salmonella enterica serovar Typhimurium. J Bacteriol.

[15] Lee HJ, Kim JA, Lee MA, Park SJ, Lee KH (2013). Regulation of haemolysin (VvhA) production by ferric uptake regulator (Fur) in Vibrio vulnificus: repression of vvhA transcription by Fur and proteolysis of VvhA by Fur-repressive exoproteases. Mol Microbiol.

[16] Pohl, E. *et al*. Architecture of a protein central to iron homeostasis: crystal structure and spectroscopic analysis of the ferric uptake regulator. *Mol Microbiol* **47**, 903–915, doi:3337 (2003). 10.1046/j.1365-2958.2003.03337.x 12581348

[17] Butcher J, Sarvan S, Brunzelle JS, Couture JF, Stintzi A (2012). Structure and regulon of Campylobacter jejuni ferric uptake regulator Fur define apo-Fur regulation. Proc Natl Acad Sci USA.

[18] Pecqueur L (2006). Structural changes of Escherichia coli ferric uptake regulator during metal-dependent dimerization and activation explored by NMR and X-ray crystallography. J Biol Chem.

[19] Traore DA (2006). Crystal structure of the apo-PerR-Zn protein from Bacillus subtilis. Mol Microbiol.

[20] Makthal N (2013). Crystal structure of peroxide stress regulator from streptococcus pyogenes provides functional insights into the mechanism of oxidative stress sensing. Journal of Biological Chemistry.

[21] Lucarelli D (2007). Crystal structure and function of the zinc uptake regulator FurB from Mycobacterium tuberculosis. Journal of Biological Chemistry.

[22] An YJ (2009). Structural basis for the specialization of Nur, a nickel-specific Fur homolog, in metal sensing and DNA recognition. Nucleic Acids Research.

[23] Dian C (2011). The structure of the Helicobacter pylori ferric uptake regulator Fur reveals three functional metal binding sites. Molecular microbiology.

[24] Sheikh MA, Taylor GL (2009). Crystal structure of the Vibrio cholerae ferric uptake regulator (Fur) reveals insights into metal co-ordination. Molecular microbiology.

[25] Bagg A, Neilands JB (1987). Ferric uptake regulation protein acts as a repressor, employing iron (II) as a cofactor to bind the operator of an iron transport operon in Escherichia coli. Biochemistry.

[26] Ernst FD (2005). Iron-responsive regulation of the Helicobacter pylori iron-cofactored superoxide dismutase SodB is mediated by Fur. Journal of bacteriology.

[27] Delany I, Spohn G, Rappuoli R, Scarlato V (2001). The Fur repressor controls transcription of iron-activated and -repressed genes in Helicobacter pylori. Molecular Microbiology.

[28] Carpenter BM (2013). Identification and characterization of novel helicobacter pylori apo-Fur-Regulated target genes. Journal of Bacteriology.

[29] Grifantini R (2003). Identification of iron-activated and -repressed Fur-dependent genes by transcriptome analysis of Neisseria meningitidis group B. Proceedings of the National Academy of Sciences of the United States of America.

[30] Gonzalez de Peredo A, Saint-Pierre C, Latour JM, Michaud-Soret I, Forest E (2001). Conformational changes of the ferric uptake regulation protein upon metal activation and DNA binding; first evidence of structural homologies with the diphtheria toxin repressor. J Mol Biol.

[31] Arnold K, Bordoli L, Kopp J, Schwede T (2006). The SWISS-MODEL workspace: a web-based environment for protein structure homology modelling. Bioinformatics.

[32] van Vliet AH, Baillon ML, Penn CW, Ketley JM (1999). Campylobacter jejuni contains two fur homologs: characterization of iron-responsive regulation of peroxide stress defense genes by the PerR repressor. J Bacteriol.

[33] Dian C (2011). The structure of the Helicobacter pylori ferric uptake regulator Fur reveals three functional metal binding sites. Mol Microbiol.

[34] Palyada, K., Threadgill, D. & Stintzi, A. Iron Acquisition and Regulation in Campylobacter jejuni. *Society* **186**, 10.1128/JB.186.14.4714 (2004). 10.1128/JB.186.14.4714-4729.2004 PMC438614 15231804

[35] Pérard J (2016). Quaternary Structure of fur Proteins, a New Subfamily of Tetrameric Proteins. Biochemistry.

[36] Gilston B (2014). a. *et al*. Structural and Mechanistic Basis of Zinc Regulation Across the E. coli Zur Regulon. PLoS biology.

[37] Baichoo, N. & Helmann, J. D. Recognition of DNA by Fur : a Reinterpretation of the Fur Box Consensus Sequence. *Society* **184**, 10.1128/JB.184.21.5826 (2002). 10.1128/JB.184.21.5826-5832.2002 PMC135393 12374814

[38] Deng Z (2015). Mechanistic insights into metal ion activation and operator recognition by the ferric uptake regulator. Nature communications.

[39] Ma Z, Faulkner MJ, Helmann JD (2012). Origins of specificity and cross-talk in metal ion sensing by Bacillus subtilis Fur. Molecular microbiology.

[40] Gilbreath JJ (2013). Random and site-specific mutagenesis of the Helicobacter pylori ferric uptake regulator provides insight into Fur structure-function relationships. Molecular Microbiology.

[41] Ma Z, Gabriel SE, Helmann JD (2011). Sequential binding and sensing of Zn(II) by Bacillus subtilis Zur. Nucleic Acids Res.

[42] Carpenter BM (2010). Mutagenesis of conserved amino acids of Helicobacter pylori fur reveals residues important for function. J Bacteriol.

[43] Sarvan S, Couture JF (2017). Method for the Successful Crystallization of the Ferric Uptake Regulator from Campylobacter jejuni. Methods Mol Biol.

[44] Reid AN, Pandey R, Palyada K, Naikare H, Stintzi A (2008). Identification of Campylobacter jejuni genes involved in the response to acidic pH and stomach transit. Applied and environmental microbiology.

[45] Tong S, Porco A, Isturiz T, Conway T (1996). Cloning and molecular genetic characterization of the Escherichia coli gntR, gntK, and gntU genes of GntI, the main system for gluconate metabolism. Journal of Bacteriology.

[46] Adams PD (2010). PHENIX: a comprehensive Python-based system for macromolecular structure solution. Acta Crystallographica.

[47] Bricogne, G. *et al*. (Global Phasing Ltd., Cambridge, 2011).

[48] Emsley P, Lohkamp B, Scott WG, Cowtan K (2010). Features and development of Coot. *Acta* Crystallographica. Section D: Biological Crystallography.

[49] Chen VB (2010). MolProbity: All-atom structure validation for macromolecular crystallography. Acta Crystallographica Section D: Biological Crystallography.

[50] Lovell SC (2003). Structure validation by Calpha geometry: phi,psi and Cbeta deviation. Proteins.

[51] Askoura M, Sarvan S, Couture JF, Stintzi A (2016). The Campylobacter jejuni Ferric Uptake Regulator Promotes Acid Survival and Cross-Protection against Oxidative Stress. Infect Immun.

[52] Deng Z (2015). Mechanistic insights into metal ion activation and operator recognition by the ferric uptake regulator. Nat Commun.

[53] Gilston BA (2014). Structural and mechanistic basis of zinc regulation across the E. coli Zur regulon. PLoS Biol.

