# Supplementary material for: Functional insights into the interplay between DNA interaction and metal coordination in ferric uptake regulators
Source: Sci Rep. 2018 May 8;8:7140. doi: 10.1038/s41598-018-25157-6 (PMC5940780; doi:10.1038/s41598-018-25157-6)
Supplement: Supplementary file 1 — Supplementary Information [file 41598_2018_25157_MOESM1_ESM.pdf]

Supplemental information for the manuscript:

Functional insights into the interplay between DNA interaction and metal coordination in ferric uptake regulators.

Sabina Sarvan[1], François Charih[1], Momen Askoura[1,2], James Butcher[1], Joseph S. Brunzelle[3], Alain Stintzi[1] and Jean-François Couture[1]

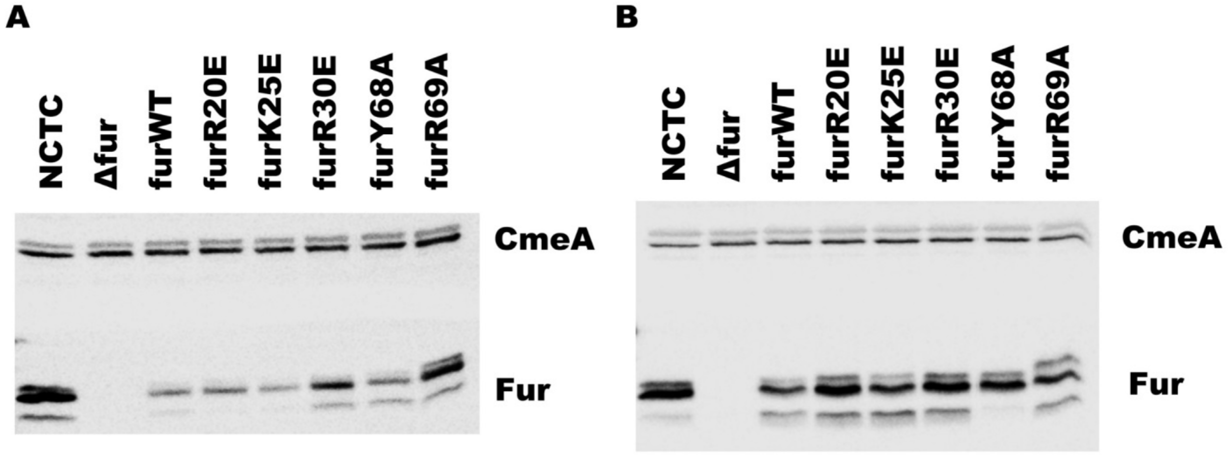

Figure S1. Expression of Fur protein in complemented *Campylobacter jejuni fur* mutants in MEM-α medium under iron poor (A) and iron replete (B) conditions. CmeA was used as loading control.

Table S1. List of primers used for cloning in this study			
**Name**	**Description**	**Primer sequence**	**Target Vector**
**Primer 1**	*Cj*Fur_Nter_NcoI	AGCAGGATCCATGGGGATGCTGATAGAAAATGTG	pStrep
**Primer 2**	*Cj*Fur_Cter_XhoI	CTATCTCGCTCGAGTTATATTTTTACCTTTGCTTTTTGA	pStrep
**Primer 3**	*Cj*Fur_Nter	GATTTAGATGTCTAGCATGCTAGTGAAAAGTTGCAAGA	pRRK
**Primer 4**	*Cj*Fur_Cter	GGGGAAGCTTTCTAGGCTTTTTCTATTCTTTGCTGCTC	pRRK

Table S2. List of primers used for EMSA in this study	
**Name**	**Primer sequence**
CjkatA60_For_1588 (Cy5 5')	ACTGAATAATTGCATTTTATTGATAATAAATTTCAAAATAAATTTAGTTTTTTTATATTA
CjkatA60_Rev_1589 (Cy5 5')	TAATATAAAAAAACTAAATTTATTTTGAAATTTATTATCAATAAAATGCAATTATTCAGT
*Cj*1345c_For_1692 (Cy5 5')	GGGAAAAGAGCCAAATTTAGGTGTTATCATCAA
*Cj*1345c_Rev_1693	TTTAACTCCTTAAATACTCTCTTGTTTTAA

Table S3. List of primers used for site-directed mutagenesis

Mutant	Name	Sequence (5'➔3')
**Arg14Glu**	*Cj*FurR14E-Forward	GTGGAATATGATGTTTTACTTGAGGAATTTAAAAAAATATTAAGACAAGGC
	*Cj*FurR14E-Reverse	GCCTTGTCTTAATATTTTTTTAAATTCCTCAAGTAAAACATCATATTCCAC
**Lys17Glu**	*Cj*FurK17E-Forward	GATGTTTTACTTGAGAGATTTAAAGAAATATTAAGACAAGGCGGACTTAAA
	*Cj*FurK17E-Reverse	TTTAAGTCCGCCTTGTCTTAATATTTCTTTAAATCTCTCAAGTAAAACATC
**Arg20Glu**	*Cj*FurR20E-Forward	CTTGAGAGATTTAAAAAAATATTAGAACAAGGCGGACTTAAATATACTAAG
	*Cj*FurR20E-Reverse	CTTAGTATATTTAAGTCCGCCTTGTTCTAATATTTTTTTAAATCTCTCAAG
**Lys25Glu**	*Cj*FurK25E-Forward	AAAATATTAAGACAAGGCGGACTTGAATATACTAAGCAAAGAGAAGTGCTT
	*Cj*FurK25E-Reverse	AAGCACTTCTCTTTGCTTAGTATATTCAAGTCCGCCTTGTCTTAATATTTT
**Lys28Glu**	*Cj*FurK28E-Forward	AGACAAGGCGGACTTAAATATACTGAACAAAGAGAAGTGCTTTTAAAAACT
	*Cj*FurK28E-Reverse	AGTTTTTAAAAGCACTTCTCTTTGTTCAGTATATTTAAGTCCGCCTTGTCT
**Arg30Glu**	*Cj*FurR30E-Forward	GGCGGACTTAAATATACTAAGCAAGAAGAAGTGCTTTTAAAAACTCTTTAT
	*Cj*FurR30E-Reverse	ATAAAGAGTTTTTAAAAGCACTTCTTCTTGCTTAGTATATTTAAGTCCGCC
**Tyr68Ala**	*Cj*FurY68A-Forward	TTAAATGTAGGAATTGCAACTGTTGCGCGTACTTTAAATTTGCTTGAAG
	*Cj*FurY68A-Reverse	TTCTTCAAGCAAATTTAAAGTACGCGCAACAGTTGCAATTCCTACATTTAA
**Arg69Glu**	*Cj*FurR69E-Forward	AATGTAGGAATTGCAACTGTTTATGAAACTTTAAATTTACTTGAAGAAGCA
	*Cj*FurR69E-Reverse	TGCTTCTTCAAGTAAATTTAAAGTTTCATAAACAGTTGCAATTCCTACATT
**ΔS2**	*Cj*FurΔS2-H43A-Forward	AAAACTCTTTATCACAGTGATACTGCCTACACACCCGAAAGTTTATATATG
	*Cj*FurΔS2-H43A-Reverse	CATATATAAACTTTCGGGTGTGTAGGCAGTATCACTGTGATAAAGAGTTTT
	*Cj*FurΔS2-H102A-Forward	CTTGCCAATAAACCTCACCATGATGCCATGATATGTAAAAATTGCGGAAAA
	*Cj*FurΔS2-H102A-Reverse	TTTTCCGCAATTTTTACATATCATGGCATCATGGTGAGGTTTATTGGCAAG
**ΔS3**	*Cj*FurΔS2-H99A-Forward	AAATACGAGCTTGCCAATAAACCTGCCCATGATCATATGATATGTAAAAAT
	*Cj*FurΔS2-H99A-Reverse	ATTTTTACATATCATATGATCATGGGCAGGTTTATTGGCAAGCTCGTATTT
	*Cj*FurΔS2-H137A-Forward	GAACATGGTTTTAAACTTACAGGGGCCTTGATGCAGCTTTATGGTGTTTGT
	*Cj*FurΔS2-H137A-Reverse	ACAAACACCATAAAGCTGCATCAAGGCCCCTGTAAGTTTAAAACCATGTTC

**Table S4. List of primers used for RT-qPCR analysis**		
**Gene**	**Primer Name**	**Sequence**
cfrA	cfrA_Forward	TCTATCAGTTTGCGCCATTG
	cfrA_Reverse	ATCAACGCCTGGGATATCTG
Cj0948c	Cj0948c_Forward	ACAAGTGGTTCTGTTGCAGT
	Cj0948c_Reverse	TTCATTCGCCTTTTGTGAGC
Cj1345c	Cj1345c_Forward	GCGTAGGAGAAAATGGAAAAA
	Cj1345c_Reverse	AAAAGCTAAATTTGGAGCCACT
fur	fur_Forward	CATTTCTTTTGGTTCAGCAGGT
	fur_Reverse	AAGCTGCATCAAATGCCCT
katA	katA_Forward	CTTTAGTCCAAGCAATATCGTTCC
	katA_Reverse	CAGCGACATTGTAAGTATTCACTTC
slyD	slyD_Forward	TACGATGAAAATGCCGTTCA
	slyD_Reverse	TTCGCCAAAAAGCTCCATAC
